# Supplementary material for: Chromothripsis during telomere crisis is independent of NHEJ, and consistent with a replicative origin
Source: Genome Res. 2019 May;29(5):737–49. doi: 10.1101/gr.240705.118 (PMC6499312; doi:10.1101/gr.240705.118)
Supplement: Supplemental Material [file supp_gr.240705.118_Supplemental_file_1.zip › contigs/annotated_contigs/DB78/contig.2.DB78_length_501_mean_cov_16.0099800399.docx]

**DB78_length_501_mean_cov_16.0099800399**

```
TATATGGGAAGGCAGAGGAACTATAGTAGTTAAAACAATTTTGAAGAGAAGAATACAATGAAAGGCATCGGTCTACCTGATTTCAAGAC
 >chr1:192994939-192995165 + E=5e-116
TTAGAGTTACAGTAATCAACACAATGTAGTACTGGCAGAGGAACAGATAAATGGAACAATGGAACAATGGACAGACTAGTGAGCCCAGA

AATAGACCCACACCAATATGCTCCATTTTTGCAAAGGTGCAGGAGGAATTCAG|TGG|TGAGTGCTTCCTTTGGATAGAGTAAAAAGTT
                                                      >chr1:193721583-193721853 + E=6e-
ATATATCTGTAGACAAATGAAGTAATTCTCTCTACTCAGTTAAAAAAGAAGAAAGAATGTATATCTTATTCTATTTGCTCATTGCAGAT
151
GAAATCAGGCATTCTCCTATCTCTTTAATAATGTATAGACTGTACAATGTCATGGATTCTGAGGCAGTGTTTGGTAAATTAATGAAAGT

GTAAGCTTTAGCTCTACTTCTGTAAAAATTAAATGTGGAATTCAATAGAGTGTGTCTA
```
